# Clinical evidence of exercise intervention in improving adults with type 2 diabetes mellitus and frailty: a narrative literature review

**DOI:** 10.3389/fphys.2026.1791522

**Published:** 2026-05-07

**Authors:** Xiuli Mo, Zhengyu Duan, Weihong Zhang, Yongliang Jia

**Affiliations:** Henan Institute of Medical and Pharmaceutical Sciences, Zhengzhou University, Zhengzhou, Henan, China

**Keywords:** exercise, frailty, inflammaging, insulin resistance, type 2 diabetes mellitus

## Abstract

**Background:**

The global aging process is accelerating with the increasing prevalence of diabetes mellitus in the elderly population. Frailty, a clinical syndrome closely related to age, is particularly prevalent in elderly with type 2 diabetes mellitus (T2DM). Previous studies indicated that some common mechanisms and exercise interventions may be an effective intervention for T2DM and frailty management.

**Objective:**

This narrative literature review aimed to provide evidence to explore possible common mechanisms and the role of exercise in management of T2DM combined with frailty.

**Methods:**

PubMed was searched for mechanistic studies. PubMed and China National Knowledge Infrastructure were searched for randomized controlled trials (RCTs) exploring exercise for T2DM and frailty.

**Results:**

Mechanistic analysis on 33 studies identified overlapping pathophysiological pathways between T2DM and frailty including encompassing inflammaging, insulin resistance, β-cell dysfunction, mitochondrial impairment, and gut dysbiosis. Evidence synthesized from 20 RCTs demonstrates that multicomponent exercise interventions could reduce frailty, lower blood glucose levels, and improve muscle strength for patients with T2DM and frailty, potentially by modulating these shared pathways, and resistance training enhances insulin sensitivity and muscle synthesis via GLUT-4 upregulation and Akt/mTOR activation.

**Conclusion:**

Multicomponent exercise and resistance training would be efficacy for elderly patients with T2DM and frailty through modulating the overlapping pathophysiological mechanisms.

## Highlights

- Shared mechanisms linking T2DM and frailty include chronic inflammation, insulin resistance, oxidative stress, metabolic dysregulation, and gut microbiota imbalance.
- Multicomponent exercise ameliorates frailty, enhances glycemic control, and improves physical function in T2DM-frailty patients by targeting shared pathophysiological mechanisms.
- Resistance training enhances insulin sensitivity and muscle anabolism by upregulating GLUT4 and activating the Akt/mTOR pathway.
- Exercise-induced AMPK activation attenuates inflammaging and senescence, disrupting T2DM-frailty pathological interplay.
- Combined exercise and nutrition interventions may be effective while optimal protocols require further investigations.

## 1 Introduction

Diabetes is one of the most common chronic diseases globally that is estimated that over 1.3 billion people will have diabetes by 2050 at the current growth rate (Ong et al., 2023). This increase is mainly due to type 2 diabetes mellitus (T2DM), which accounts for over 90% of all diabetes cases. The prevalence of T2DM rises with age, exceeding 20% in those aged 65 and older, with the highest rate of 24% in the 75–79 age group (Sun et al., 2022). Population aging, while reflecting medical and living standard improvements, also increases the diabetes-related medical burden, especially for elderly T2DM patients. With T2DM duration increases, the risk of hypoglycemia and cardiovascular complications for T2DM patients rises significantly. What is worse, T2DM patients in advanced age have higher physical frailty than younger ones (Huang et al., 2014).

Frailty, an emerging global burden, impacts clinical practice and healthcare spending worldwide. It is considered an age-related clinical condition, typically marked by reduced physiological capacity across multiple organ systems and increased vulnerability to stressors (ElsaDent et al., 2019). This high vulnerability is due to changes in several physiologic systems, mainly inflammation, insulin-resistance (IR), alterations in coagulation systems, endothelial and vascular dysfunction (Angulo et al., 2016). The frailty phenotype is identified by five criteria: weakness, slow gait speed, low physical activity, exhaustion, and unintentional weight loss. Frailty states are classified as “frail” with three or more deficits, “pre-frail” with one or two deficits, and “non-frail” with no deficits (Hoogendijk et al., 2019).

There is a complex bidirectional relationship between T2DM and frailty, with each influencing the other and potentially creating a vicious cycle. In a cross-sectional study in primary health care settings in 3 semi-urban regions in Greece, compared with their peers without diabetes, individuals with T2DM aged ≥65 years show higher vulnerability in cognitive and physical performance, especially in the older age group of 65–74 years (Kotsani et al., 2018). Frailty can also aggravate T2DM. Fragile patients are significantly less physically active, and the lack of muscle movement makes glucose uptake and metabolism less efficient, making it more difficult to keep blood glucose in the normal range. On the one hand, IR in T2DM impairs physical function and mobility, increases the risk of frailty, and is a significant risk factor accelerating the progression from pre-frail to frail. On the other hand, frailty may increase the risk of pathological fractures (Nishikawa et al., 2021).

The coexistence of T2DM and frailty in older adults is not surprising, as these two age-related diseases share common underlying pathophysiologic mechanisms. Common mechanisms for T2DM and frailty mainly include chronic inflammation, insulin resistance, oxidative stress, and metabolic disorders, all of which play critical roles in both. Considering the high prevalence of T2DM among frail elderly individuals, as well as the complexity of the disease and the burden of its associated complications, it is essential to identify this vulnerable patient population who require close follow-up.

Notably, regarding targeted exercise interventions, a network meta-analysis by Qin et al. (Qin et al., 2024) demonstrated that multicomponent exercise offers significant comprehensive advantages in reducing fasting blood glucose(FBG) and enhancing physical function, while Pilates training was identified as the optimal intervention for lowering HbA1c and postprandial glucose levels. These clinical findings further underscore the importance of diverse exercise modalities in the synergistic regulation of glucose and lipid metabolic homeostasis. This identification could facilitate the implementation of therapeutic measures and intervention strategies to prevent further deterioration of their functional status (Assar et al., 2019).

Given these complex and overlapping mechanisms and the growing clinical evidence from network meta-analyzes, we designed the present article as a narrative literature review to complement the work by Qin et al. Rather than comparing the relative efficacy of specific exercise modalities, we conducted a structured search and narratively synthesized (1) the shared pathophysiological pathways linking T2DM and frailty and (2) the effects of different exercise modalities and exercise-based multidomain lifestyle programs on frailty, glycemic control, and physical function in older adults with comorbid T2DM and frailty.

## 2 Mechanisms

### 2.1 Mechanism leading to T2DM

T2DM is one of the most common metabolic disorders globally. Its development primarily results from the interplay of two key factors: impaired insulin secretion by pancreatic β-cells and IR in insulin-sensitive tissues (Roden and Shulman, 2019). Insulin release and its metabolic effects must be precisely regulated to meet metabolic demands and maintain glucose homeostasis. Therefore, the molecular mechanisms involved in insulin synthesis, secretion, and action, as well as the feedback regulation between insulin-sensitive tissues and β-cells, must function optimally. Any defect or dysregulation in these processes can lead to metabolic imbalance and contribute to the pathogenesis of T2DM. In the context of β-cell dysfunction, reduced insulin secretion impairs the body’s ability to maintain normal blood glucose levels. Meanwhile, IR leads to increased hepatic glucose production and decreased glucose uptake in skeletal muscle, liver, and adipose tissue. Although both processes often occur early in disease development and drive the progression of T2DM, β-cell dysfunction typically has a more detrimental impact on metabolic balance than IR alone. However, when β-cell dysfunction and IR coexist, hyperglycemia is exacerbated, accelerating the progression of T2DM. This dual mechanism is a critical factor in the worsening of hyperglycemia during the disease process (Galicia-Garcia et al., 2020; Zheng et al., 2017).

#### 2.1.1 Insulin resistance

IR, a metabolic dysfunction that reciprocally interacts with immune responses, particularly chronic inflammation, refers to impaired responsiveness of muscle, liver, and adipose tissues to physiological insulin levels within the perspective of immunology. So, the body needs more insulin than usual to keep blood sugar in check. Normally, insulin has key roles in regulating glucose: it suppresses hepatic glucose production (HGP), slows down fat breakdown, helps cells absorb glucose from the blood, and promotes net glycogen synthesis. But in IR tissues, these functions don’t work properly even when insulin levels are normal (Petersen and Shulman, 2018).

Nowadays, studies have revealed two major insulin signaling pathways: the phosphatidylinositol 3-kinase/protein kinase B (PI3K/Akt) pathway and the Ras/mitogen-activated protein kinase (RAS/MAPK) pathway (Ye et al., 2022). The PI3K/Akt pathway is the classic mechanism in insulin signaling. PI3K is the core enzyme of this pathway and consists of the catalytic subunit p110 and the regulatory subunit p85. Its main function is to catalyze the conversion of phosphatidylinositol (4,5)-bisphosphate (PIP2) to phosphatidylinositol (3,4,5)-trisphosphate (PIP3), and the generation of PIP3 is a key signaling step, which activates the downstream Akt protein kinase via phosphatidylinositol-dependent kinase 1 (PDPK1) to initiate a series of metabolic and physiological processes in the cell (Saltiel, 2021).

Imbalance of insulin signaling is an important mechanism of IR, which is manifested in two main aspects: inactivation of insulin receptor substrate (IRS) proteins and dysfunction of the insulin signaling pathway (Fan et al., 2024). Inactivation of IRS proteins may be caused by alterations in insulin receptor structure, number, or binding affinity, resulting in a reduction in signaling capacity. Dysfunction of the insulin signaling pathway is manifested by abnormalities in key nodes such as PI3K and Akt, including insufficient phosphorylation, abnormal distribution, or aberrant expression, which impede signaling. These abnormalities ultimately lead to insulin’s inability to effectively regulate metabolic processes, triggering IR.

#### 2.1.2 β-cell failure

β-cell failure represents an intrinsic cellular dysfunction capable of indirectly eliciting inflammatory responses. In an IR state, pancreatic β-cells respond to increased metabolic demand by compensatory increases in insulin secretion. However, a state of persistent nutritional overload and insulin resistance triggers a vicious cycle: prolonged hyperinsulinemia not only leads to insulin receptor desensitization, but also exacerbates the degree of IR (Cui et al., 2024). When the compensatory mechanisms of the β-cells are unable to respond effectively to the continuously increasing metabolic stress, the cells will breach the physiological threshold of compensation and enter a pathological state. This loss of compensation is accompanied by a decrease in the number of β-cells and functional deterioration, which ultimately triggers an irreversible phase of insufficient insulin secretion and becomes a critical turning point in the development of T2DM.

The mechanisms of functional pancreatic β-cell failure are threefold: first, a decrease in the number of β-cells, as evidenced by an increase in histologically confirmed β-cell apoptosis; second, β-cell dysfunction, in which prolonged metabolic loading results in the inability of the β-cells to respond properly to glucose stimulation and abnormal insulin secretion; and finally, a loss of β-cell identity, as evidenced by dedifferentiation, which results in a loss of their specific functional properties. Together, these mechanisms ultimately lead to a total decline in β-cell function (Niu et al., 2024). This process is often closely linked to factors such as metabolic stress, inflammation and oxidative stress.

#### 2.1.3 Genetic effects and lifestyle influences

The pathogenesis of T2DM involves a complex interplay of genetic and lifestyle factors. Lifestyle-related risk factors, including unhealthy energy-dense dietary patterns, physical inactivity, chronic stress, and the resultant obesity, significantly increase the susceptibility to T2DM development (Chatterjee and Davies, 2017). These factors further exacerbate the onset and progression of the disease by affecting insulin sensitivity and metabolic function.

##### 2.1.3.1 Genetic effects

Genetic susceptibility to T2DM plays an important role in the development of the disease, with heritability ranging from approximately 30%-70% (DeForest and Majithia, 2022). This range is wide because of differences in different study populations, the influence of environmental factors, and the complex interactions between genes and the environment. Studies have shown that a person’s risk of developing T2DM roughly doubles if they have a sibling with the disease and triples if they have a parent or child with T2DM (DeForest and Majithia, 2022). This increased risk is not only due to genetic patterns, but is also closely linked to lifestyle habits and environmental influences shared by family members.

A study of 650 middle-aged adults from the NUTRIHEP cohort (Southern Italy) linked the *FOXO3* rs2802292 polymorphism to T2DM. The TT genotype was associated with higher diabetes prevalence and a 2.14-fold increased T2DM risk compared to GG (OR = 2.14, 95% CI: 1.01-4.53, p = 0.05), while the G allele conferred protection (OR = 0.45, 95% CI: 0.25-0.81, p = 0.008). Mechanistically, the G allele enhanced *FOXO3* expression and insulin sensitivity, whereas the TT genotype reduced *FOXO3* and promoted insulin resistance. Thus, *FOXO3* rs2802292 is a significant genetic marker for T2DM, with TT as a risk genotype and G as a protective allele (Forte et al., 2024).

APOE ϵ4 carriership was associated with significantly higher risk of frailty (OR = 2.75, 95% CI: 1.38-5.50, p = 0.004), demonstrating a dose-dependent effect, in a study of 1,234 older adults from the HELIAD cohort. Frailty was defined by ≥3 modified Fried criteria. The association remained significant after excluding dementia cases and specific genotypes, suggesting APOE ϵ4 as an independent genetic biomarker for frailty, potentially mediated by β-amyloid and oxidative stress (Mourtzi et al., 2019).

##### 2.1.3.2 Lifestyle influences

Lifestyle choices also play a crucial role in the development of T2DM. Unhealthy eating habits, such as dietary patterns high in sugar, fat, and salt, not only lead to obesity, but also directly impair pancreatic β-cells function, affecting insulin secretion and action. At the same time, energy-dense diets such as fast food and processed foods increase the accumulation of body fat, further aggravating IR.

Jianfan Zhou et al.’s cohort study has demonstrated that a pro-inflammatory diet is associated with an increased risk of T2DM (Zhou et al., 2024). Specifically, this type of diet can elevate the circulating levels of pro-inflammatory cytokines such as interleukin-6 (IL-6) and C-reactive protein (CRP), thereby inducing chronic systemic inflammation; simultaneously, it may further augment the risk of T2DM by exacerbating insulin resistance; furthermore, a pro-inflammatory diet can disrupt glucose metabolic homeostasis, ultimately increasing the susceptibility to T2DM.

A sedentary lifestyle, on the other hand, weakens the body’s sensitivity to insulin by reducing energy expenditure and muscle uptake of glucose, thus increasing the risk of T2DM.

#### 2.1.4 Adipose tissue dysfunction

Several age-related changes in adipose tissue are considered endogenous contributors to metabolic dysfunction (Ou et al., 2022). Adipocyte hypertrophy is accompanied by inefficient nutrient transport and impaired cellular signaling, leading to metabolic impairments and reduced energy expenditure (Von Bank et al., 2021). A strong association exists between adipose tissue dysfunction and chronic inflammation induced by pro-inflammatory factor secretion (Chen and Nuñez, 2010). Lipid redistribution and chronic inflammation trigger metabolic perturbations, encompassing IR, impaired glucose tolerance, and diabetes. In dysfunctional adipose tissue, elevated pro-inflammatory cytokines may directly disrupt insulin signaling pathways (Stienstra et al., 2010). Moreover, age-associated immune cell modifications, such as T-cell accumulation, represent potential contributors to IR (Bapat et al., 2015).

### 2.2 Mechanisms for frailty

The pathophysiologic mechanism of frailty, a complex clinical syndrome, involves a progressive decline in multi-system functioning. Studies have shown that the accelerated aging process at the subcellular and cellular levels encompasses the core biological mechanisms of chronic inflammation, loss of cell proliferation with secretion of pro-inflammatory factors, mitochondrial dysfunction, and dysregulation of nutrient-sensing signaling pathways that regulate growth and metabolism (Kim et al., 2024). These pathological changes associated with aging interact with each other through a complex molecular network, and the functional decline and coordination of multiple physiological systems are abnormal, ultimately manifesting in the clinical manifestations as a debilitating syndrome characterized by muscle atrophy, decreased physical strength, lowered immunity, and weakened multiorgan reserve function.

#### 2.2.1 Inflammaging and cellular senescence

With aging, a slow but persistent pathological state characterized by chronic low-grade inflammation emerges. In immunology, this sterile inflammatory condition derived by dysregulated immune homeostasis and persistent innate immune activation is termed *inflammaging*. (Franceschi et al., 2018). Cellular senescence is a stable and irreversible state of terminal growth arrest. Even under ideal external conditions, such as adequate nutrition and growth factors, and when stimulated by signals that promote division, cells are unable to initiate DNA replication and division programs and remain in a non-proliferative state (Di Micco et al., 2020). Senescent cells drive the aging of organs and the body through a variety of mechanisms. Further exacerbating the body’s inflammatory response can lead to a decline in the body’s immune function, which creates a vicious cycle that can trigger frailty (Li et al., 2023).

#### 2.2.2 Mitochondrial dysfunction

As a form of cellular organelle dysfunction that can indirectly activate immune responses, mitochondrial dysfunction is another key mechanism closely associated with the development of frailty, the causes of which include mitochondrial DNA mutations, respiratory chain complex instability, and mitochondrial homeostatic imbalance (Kim et al., 2024). This set of abnormalities leads to increased reactive oxygen species chemistry (ROS), decreased ATP production, and increased mitochondrial damage, resulting in inflammation and cell death (Baechle et al., 2023; Lopez-Otin et al., 2023).

#### 2.2.3 Reduced muscle mass

One of the most obvious manifestations of frailty individuals is a decrease in muscle strength. Aging disrupts the homeostasis of skeletal muscle, leading to an imbalance between the anabolic and catabolic pathways of muscle proteins, resulting in a loss of overall skeletal muscle mass, which is manifested mainly by atrophy and a decrease in the number of type II muscle fibers, as well as an abnormal infiltration of intramuscular and intermuscular fat (Cruz-Jentoft and S, 2019). The process of rising fat percentage not only releases inflammation hindering the muscle’s ability to repair itself; it also increases the metabolic burden.

### 2.3 Mechanisms common to frailty and T2DM

**Figure 1:**
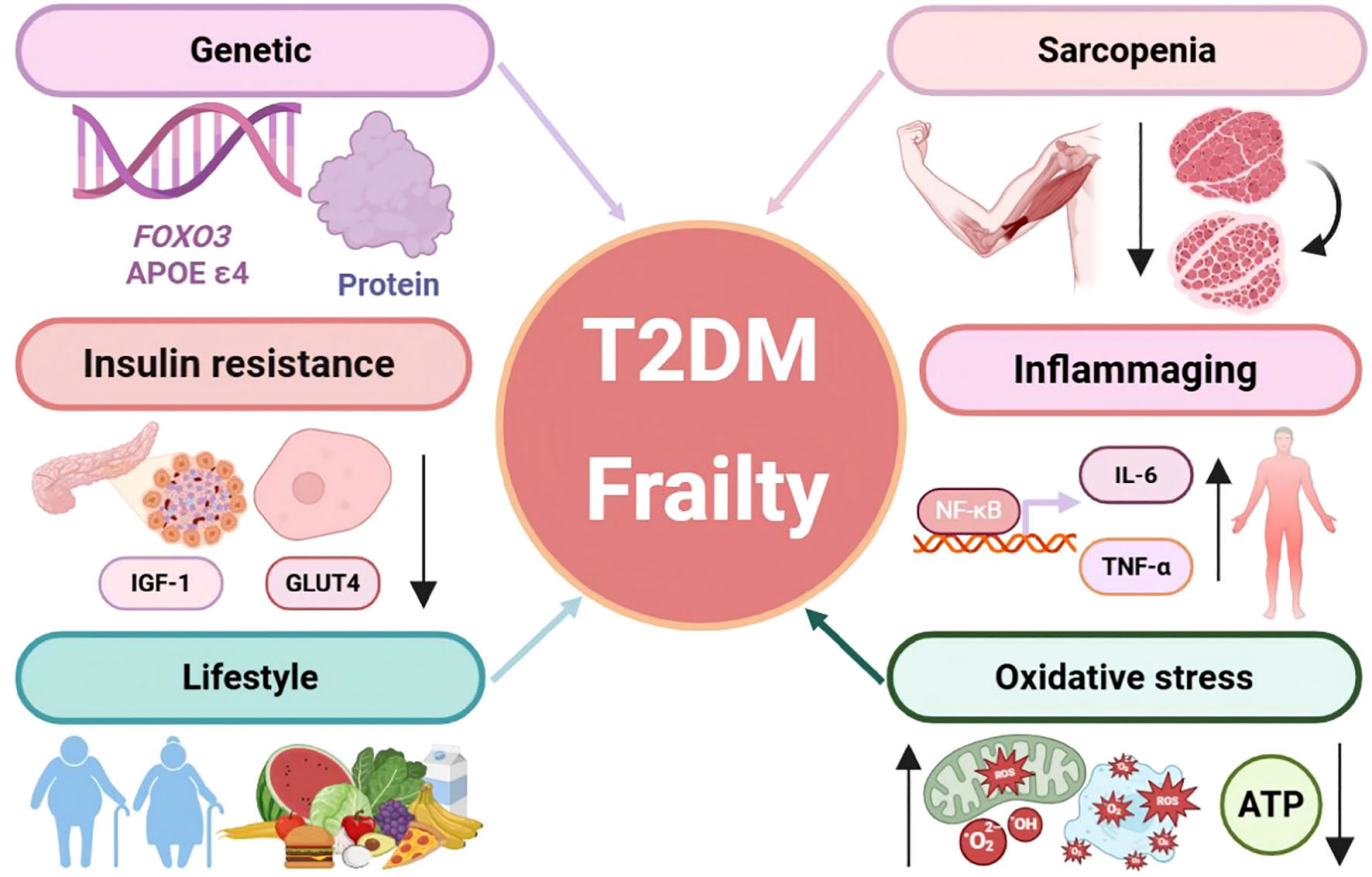
Common mechanisms linking T2DM and Frailty.

There is a high degree of overlap in the pathogenesis of T2DM and frailty, with chronic inflammaging, IR, mitochondrial dysfunction, metabolic imbalances, and disturbances in gut flora. These interconnected mechanisms form a multifactorial relationship between the two conditions **Figure 1**, providing potential therapeutic targets for multicomponent exercise interventions.

#### 2.3.1 Inflammaging

Inflammaging is characterized by chronic low-grade inflammation due to immune homeostasis imbalance and reflects persistent activation of innate immunity in immunology. Inflammaging is an extremely critical factor, and prolonged exposure to IL-6 signaling may directly mediate muscle catabolism; there is a significant correlation between levels of CRP and muscle strength and mass; and another pro-inflammatory factor, TNF-α, can act directly on skeletal muscle and affect muscle content (Tuttle et al., 2020).

#### 2.3.2 Mitochondrial dysfunction and oxidative stress

In T2DM, IR is characterized by impaired glucose uptake by peripheral tissues. In frailty, IR inhibits glucose and lipid metabolism by decreasing muscle and hepatic responsiveness to insulin, leading to a decrease in the efficiency of mitochondrial oxidative phosphorylation, which triggers a lack of ATP production and an accumulation of ROS, a process that not only exacerbates pancreatic islet β-cells impairment but also accelerates the process of skeletal muscle atrophy through oxidative stress.

#### 2.3.3 Metabolic imbalance

Metabolic imbalances contribute to the development of T2DM and frailty. AMP-activated protein kinase (AMPK) is an evolutionarily conserved serine/threonine kinase that plays a central role in maintaining cellular metabolic homeostasis (Rabinovitch et al., 2017). When the AMPK pathway is over-activated in patients with frailty and T2DM, fatty acid oxidation in T2DM increases the lipotoxic while AMPK in frailty accelerates muscle catabolism, gradually leading to metabolic imbalance.

#### 2.3.4 Intestinal flora imbalance

Gut dysbiosis, a microecological perturbation intimately linked to immune modulation, plays a pivotal role in metabolic diseases. Notably, the gut microbiome harbors a genetic reservoir exceeding the human genome by an order of magnitude (Fan and Pedersen, 2020). Severalxstudies have shown a correlation between T2DM and changes in gut microorganisms: the diversity of gut microbiota is significantly reduced in T2DM patients, with decreased abundance of *Bifidobacterium* and *Akkermansia*, and increased abundance of *Dachshund bacilli*; patients who have undergone colon resection exhibit an increased risk of T2DM, indirectly suggesting that gut microbiota and hormone secretion from the distal intestinal tract may be involved in glucose metabolism regulation (Chen and Li Wang, 2021).

Increased levels of γ-linolenic acid were positively correlated with the risk of T2DM, and baseline levels of γ-linolenic acid were negatively correlated with the abundance and diversity of intestinal microorganisms, suggesting that intestinal microorganisms mediate the association between γ-linolenic acid and T2DM (Miao et al., 2020).

Rashidah, Nur Hannah et al. found that compared to their healthy counterparts, frail older adults had a less gut microbiota diversity, with higher numbers of *Ruminococcus, Dialister* and *Lactobacillus.* They also increased zonulin, pro-inflammatory cytokines and amino acids. In contrast, healthy older adults had more short-chain fatty acid (SCFA) producers in their bodies (Rashidah et al., 2022).

## 3 Clinical randomized controlled trials

In intervention studies for patients with T2DM combined with frailty, randomized controlled trials (RCTs) are recognized as the definitive method for validating the causal effects of interventions by virtue of their scientifically rigorous design. By randomly assigning participants and setting up a control group, RCTs are able to effectively control for confounders and bias, thereby clearly revealing a direct link between the intervention and the study outcome.

A total of 20 randomized controlled trials (RCTs) were included in this narrative literature review to synthesize the clinical evidence. A systematic literature search was conducted across the PubMed and China National Knowledge Infrastructure (CNKI) databases from their inception to June 2025. The search strategy utilized Medical Subject Headings (MeSH) combined with key terms, including “Type 2 Diabetes Mellitus,” “Frailty,” and “Exercise.” Studies were selected based on the following PICOS criteria:

1. Participants: Adults aged ≥45 years with comorbid T2DM and frailty were included. Participants requiring acute medical management were excluded.
2. Interventions: Physical activity interventions lasting ≥12 weeks, including exercise-only and exercise-based multidomain programs combining exercise with nutritional or educational support, were included.
3. Comparison: Control groups received usual care or no active intervention.
4. Outcomes: Primary outcomes included frailty status (measured by the Fried phenotype or Frailty Index) and glycemic control (HbA1c and fasting glucose). Secondary outcomes included physical function, assessed by the Short Physical Performance Battery (SPPB) score.
5. Study Design: RCTs published in English and Chinese were included.

### 3.1 Quality assessment of included RCTs

Systematic evaluation, as an important method of synthesizing evidence of the effectiveness of interventions, is the cornerstone of the construction of clinical guidelines and evidence-based medicine systems. In this study, two researchers independently evaluated the RoB for each included RCT using the Cochrane Risk of Bias Tool (RoB 2), version 2(Sterne et al., 2019). We assessed RoB in five domains: (1) the randomization process; (2) interventions that deviated from expectations; (3) missing outcome data; (4) measurement of outcomes; and (5) selection of reported outcomes. Any discrepancies in the assessment were resolved through discussion and consensus, or by consulting a third expert if necessary.

**Figure 2:**
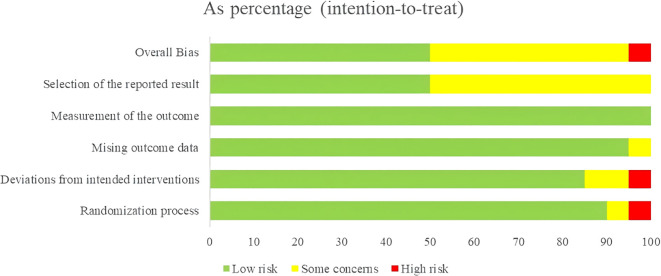
Risk of bias of included RCTs with the RoB2 tool.

Each domain’s RoB was rated as “low”, “high”, or “some concerns”. An RCT was rated as having “low” RoB if all five domains were rated “low”; it was rated “high” if at least one domain was rated “high”. If neither of the above applied, the RCT was assessed as “some concerns”. The RoB assessment results for all included RCTs are presented in **Figure 2**. A total of 1 article was judged to be high risk and 9 articles were judged to be some concerns in this assessment. Of the randomization process evaluations, 1 was judged to be high risk and 1 was rated as some concerns. Only 1 of the deviations from intended interventions was rated as high risk, while 2 were rated as some concerns. In the evaluation of missing outcome data, 1 was rated as some concerns, and the rest were low risk. All studies were assessed as low risk in the measurement of the outcome. In the selection of the reported result, 9 articles were assessed as having some concerns, with the remaining judged as low risk. The detailed “Study × Domain” evaluation is presented in the Risk of Bias Summary Figure in **Supplementary Figure S1**.

### 3.2 Characteristics of included RCTs

A total of 20 RCTs were included in this review. The detailed characteristics of these studies, including author, publication year, study design, follow-up duration, intervention type, participant age, outcome evaluation measures, and key results, are summarized in **Table 1**. The cumulative evidence from these 20 RCTs indicates that various exercise interventions yield positive outcomes, specifically contributing to the improvement of frailty status (denoted as ①), lowering blood glucose levels (denoted as ②), and enhancing physical functions (denoted as ③) in older patients with diabetes. The interventions varied across studies, including resistance training (RT), multicomponent exercise (ME), moderate-intensity aerobic walking (MAW), elastic band training (EBT), and traditional Chinese exercises (TCE) such as Baduanjin. For specific data on HbA1c changes and frailty scores in each study, please see **Supplementary Table S2**.

**Table 1:** Summary of randomized controlled trials on exercise for frailty in patients with diabetes.

Modality	Author and year	Design	Age	Interventions	Evaluation	Follow-up (month)	Results
Resistance Training (RT/EBT)	Rodriguez-Mañas et al., 2019	RCT	≥70	Multidomain lifestyle	SPPB	12	②③
	Wang Xiaohua et al., 2022	RCT	>50	Exercise+education	FBG, HbA1c, ESE	3	①②③
	Álvarez-Bustos et al., 2024	RCT	≥70	Exercise+nutrition	SPPB	4	③
	Sinclair et al., 2024	RCT	≥70	Exercise+nutrition	QOL, Barthel Index	24	①③
	Laosa et al., 2025	RCT	>70	Multidomain lifestyle	FFP, SPPB	18	①③
	Yu et al., 2019	RCT	≥60	Exercise+education	FBS, HbA1c, FI	3	①②③
	Xiuzhen, 2021	RCT	48-70	Exercise-only	FBS, HbA1c, 2hPG, BMI, FI	3	①②③
	Huang et al., 2024	RCT	>60	Exercise+nutrition	FBG, FAS	3	①②③
	Lin et al., 2025	RCT	≥60	Exercise-only	SPPB, HbA1c,	3	①②③
Multicomponent Exercise (ME)	Hu et al., 2019	RCT	≥65	Exercise+nutrition	FPG, HbA1c, FFP	3	①②③
	Zhang et al., 2020	RCT	≥60	Exercise-only	FBG, TFI, SPPB	3	①③
	Shujing, 2021	RCT	≥65	Exercise-only	TFI, HbA1c, FBG, 2hPG, SPPB	6	①②③
	Yang, 2022	RCT	≥60	Exercise-only	FP, FBG, BBS	3	①②③
	Shenju et al., 2022	RCT	≥60	Exercise-only	TFI, SPPB, FBG	3	①②③
Moderate-intensity aerobic walking (MAW)	Simpson et al., 2020	RCT	45-76	Multidomain lifestyle	FI	96	①③
	Hazuda et al., 2021	RCT	63.2 ± 9.93	Multidomain lifestyle	FFP	168	①③
	Espinoza et al., 2022	RCT	45-76	Multidomain lifestyle	FFP	168	①
	Espeland et al., 2022	RCT	45-76	Multidomain lifestyle	FI	96	①③
	Evans et al., 2023	RCT	45-76	Multidomain lifestyle	FI-E	216	①③
Traditional Chinese Exercises (TCE)	Lin, 2024	RCT	≥60	Exercise-only	TFI, SPPB	3	①②③

Evaluation.

SPPB, Short Physical Performance Battery; FI, Frailty Index; FFP, Fried Frailty Phenotype; ESE, Exercise Self-Efficacy Scale QOL, Quality of Life; FBS, Fasting Blood Sugar; HbA1c, Glycated Hemoglobin; TFI, Tilburg Frailty Indicator; FBG, Fasting Blood Glucose; 2HPG, 2-Hour Postprandial Glucose; BMI, Body Mass Index; FAS, Frailty Assessment Scale; BBS, Berg Balance Scale.

①, Frailty improvement; ②, Lower blood glucose; ③, Improved physical functions.

#### 3.2.1 T2DM status

The duration of T2DM required two years or more after diagnosis, with the longest follow-up up to 16.9 years, reflecting the need for chronic management. Glycemic control baseline HbA1c averaged 7.28%-7.33%, with most studies requiring HbA1c < 11% and fasting glucose < 126 mg/dL to exclude those with severe glycemic control. Overweight or obese people (BMI≥25 kg/m²) were also included, highlighting the co-morbidity of obesity with T2DM as well as frailty.

#### 3.2.2 Frailty status

Stratification based on the Fried Frailty phenotype: To be diagnosed with frailty, at least three of the following five conditions must be met: involuntary weight loss, that is, ≥4.5 kilograms per year; Low grip strength, less than 26 kilograms for men and less than 18 kilograms for women, stratified by gender. Slow gait speed, less than 0.8 meters per second in the 4-meter walk test; Low physical activity, less than 150 minutes of moderate-intensity exercise per week; Self-reported feeling tired. There are also studies focusing on patients with pre-frailty, that is, meeting 1 to 2 criteria, concentrating on the early reversible stage of the disease. Excluding those with a Barthel index lower than 60, the ability to independently complete basic activities is required.

### 3.3 Outcomes

#### 3.3.1 Frailty state reversal

Intervention measures through multi-system regulation can effectively reduce Frailty Index (FI), thereby improving the frailty state of the elderly. The FI is a key tool for assessing aging-related health status. The degree of defect accumulation FI is quantified by the FI, which is the number of defects present as a proportion of the total number of defects assessed (Angulo et al., 2016).

It reflects the overall degree of aging by calculating the proportion of the number of health defects an individual has to the total assessment items. Health defects include diseases, symptoms, functional abnormalities and others. The value range of FI is from 0 (no defect) to 1 (complete defect), and it is usually divided into three grades: low frailty (FI ≤ 0.10), medium frailty (0.10<FI ≤ 0.21), and high frailty (FI>0.21).

The research by Mark A. Espeland et al. pointed out that the accumulation rate of FI in the Intensive Lifestyle Intervention group (ILI) was significantly lower than that in the Diabetes Support Education group (DSE), with average annual growth rates of 0.004 and 0.012 respectively (P<0.001). After 8 years of intervention, the proportion of people with high FI in the ILI group decreased by 6.4%. Furthermore, FI was negatively correlated with the 400-meters walking speed (r=-0.42, P<0.001), indicating that the walking speed of people with high FI decreased more rapidly. It is notable that the all-cause mortality rate of the population with FI>0.21 is 2.3 times that of the population with low FI (Espeland et al., 2022).

Sara E. Espinoza et al. demonstrated that prior assignment to ILI significantly improved physical function metrics (Espinoza et al., 2022). Joni K. Evans et al. documented that over 10 years of multidomain ILI significantly reduced the deficit-accumulation frailty index (FI-E). At 18-year follow-up, the ILI group maintained a mean FI-E score 0.0130 lower than the DSE group (p<0.001). This benefit persisted both during and after the intervention period, suggesting ILI may confer long-term deceleration of biological aging as measured through FI-E (Evans et al., 2023).

Liu Shujing’s research found that after 6 months of multicomponent exercise intervention, the scores of each dimension of Tilburg Frailty Indicator (TFI) in the observation group were significantly lower than those in the control group (P<0.05), and the frailty state was significantly improved (Shujing, 2021). Zhang Shuang et al. demonstrated that multicomponent exercise can effectively improve the frailty status of elderly patients with T2DM and frailty by reducing the physical dimension score, psychological dimension score, and total score of TFI (Zhang et al., 2020). Fang et al. reported that after 12 weeks of ME, the total Tilburg Frailty Indicator score in the observation group decreased significantly from11.5 ± 2.3 to 7.5 ± 0.2, confirming the significant effect of this exercise mode in alleviating frailty in patients with T2DM (Shenju et al., 2022).

The TFI is a self-report scale assessing individual frailty, characterized by self-reported symptoms of physical, psychological, and social functioning, enabling frailty quantification via self-assessment. The Chinese version of TFI has been localized. Its Cronbach’s α coefficient in the elderly population of the community is 0.71, which has good reliability and validity and is suitable for the Chinese population (Shujing, 2021).

FI and TFI are negatively correlated with the SPPB score. That is, the higher the index values of FI and TFI, the lower the SPPB score. Among them, FI includes functional defects such as decreased muscle strength and abnormal gait, and these factors will directly affect the test performance of SPPB. After multicomponent exercise intervention, the total score of SPPB and the scores of each item have increased, while the total score of TFI and the scores of the dimensions of physical frailty and psychological frailty have decreased. This indicates that the improvement of physical function and the alleviation of frailty occur simultaneously, and the degree of improvement of the two is positively correlated.

#### 3.3.2 Decreased blood glucose levels

The Glycosylated Hemoglobin, Type A1C (HbA_1_c) compliance rate of the multicomponent exercise intervention group increased from 26% at baseline to 36.8% at 12 months (P = 0.02) (Rodriguez-Mañas et al., 2019). This improvement was significantly higher than that of the conventional care group, while the improvement of the latter was only reflected in the blood pressure compliance rate. The increase in the HbA_1_c compliance rate brought about by multicomponent exercise intervention may be related to the increase in muscle mass and the improvement of insulin sensitivity. Resistance training can improve the uptake of glucose by skeletal muscles, while nutritional education strengthens dietary compliance. Furthermore, the HbA_1_c level in the ILI group was lower than that in the DSE group, the incidence of glycemic-related complications was lower, and the growth rate of the flimsy index FI was slower (Simpson et al., 2020). This indicates that blood glucose control may have a potential role in delaying aging-related pathologies. Subgroup analysis showed that among people aged 45-59, ILI had a more significant improvement in blood glucose and FI, which might be related to the better metabolic reserve of this age group.

The research by Zeng Xiuzhen and Hu Lin et al. reveals that elastic bands have a significant effect on lowering fasting blood glucose. The research results showed that the FBG level of the subjects before the intervention was 8.92 ± 1.09 mmol/L, and it decreased to 6.63 ± 0.44 mmol/L after the intervention. Another set of data decreased from 11.02 ± 5.69 mmol/L before the intervention to 7.87 ± 1.06 mmol/L after the intervention (Lin et al., 2025; Xiuzhen, 2021). From the mechanism perspective, resistance exercise can increase the expression of GLUT-4 in skeletal muscle, effectively promote glucose uptake, and improve IR. Meanwhile, the application of elastic bands in resistance exercises can also enhance the exercise compliance of participants and ensure the continuity and stability of intervention measures.

Wang et al. found that, under the guidance of a multidisciplinary team, the FBG levels in the observation group significantly decreased from 8.52 ± 1.65 mmol/L before intervention to 6.11 ± 1.27 mmol/L after intervention, demonstrating a markedly better glycemic control effect compared to the control group. Additionally, the HbA1c levels in this group effectively improved from 7.71% ± 1.52% to 6.45% ± 1.25%, confirming the significant value of closed-loop empowerment intervention in enhancing the quality of glycemic management(Wang Xiaohua et al., 2022). Jin Yu et al. demonstrated that after three months of resistance training, the HbA1c level of patients with T2DM complicated with frailty decreased from 7.78 ± 1.19 to 6.68 ± 0.78, and this level was significantly higher than that in the control group without exercise intervention (Yu et al., 2019).

A study by Hu et al. showed that following combined nutritional and exercise intervention, significant reductions were observed in both FPG (from 8.50 ± 0.97 mmol/L to 4.36 ± 1.34 mmol/L) and HbA1c (from 8.33% ± 1.80% to 6.54% ± 1.79%) (Hu et al., 2019). Huang Xiaoni et al. found that a three-month elastic band training intervention significantly improved FBG levels measured at 3 a.m. in patients with T2DM and frailty, reducing the values from 7.78 ± 1.05 mmol/L to 6.17 ± 0.93 mmol/L (Huang et al., 2024).

Whether it is multidimensional interventions, octogenarians or resistance training, all of these measures have a positive effect on lowering blood glucose levels. In clinical practice, the selection of exercise programs should be stratified according to the degree of frailty of the patient, such as by referring to the TFI score and the results of the SPPB physical fitness test. At the same time, behavioral support should be used to enhance patients’ compliance, so as to achieve effective glycemic control under the premise of ensuring safety.

#### 3.3.3 Improvement of physical function

When the exercise intervention groups were analyzed in comparison with the control group, it was seen that the exercise intervention groups performed significantly better than the control group in all the dimensions of SPPB. The SPPB is a tool for objectively assessing the functional performance of older adults and is widely used in geriatric and epidemiologic studies to measure functional decline or improvement associated with aging and disease. At its core, the SPPB is a standardized test that comprehensively assesses lower extremity function, balance, and muscle strength, thereby reflecting the overall state of physical functioning.

The research by Leocadio Rodriguez-Manas et al. showed that the SPPB score of the intervention group was on average 0.85 points higher than that of the conventional care group, 95% CI: 0.44-1.26, P<0.0001 (Rodriguez-Mañas et al., 2019). Alejandro Álvarez-Bustos et al.’s further analysis reveals that in patients with compliance of 85% or higher, the improvement in SPPB is more significant (Álvarez-Bustos et al., 2024). Moreover, 46% of the patients in the intervention group improved by 1 point or more in SPPB, which was significantly higher than 38% in the conventional nursing group (P = 0.001). Sensitivity analysis indicated that the research results were robust. Subgroup analysis showed that the baseline frailty status (frailty/pre-frailty) did not affect the intervention effect, but age, baseline SPPB score and the number of frailty criteria would have an impact on the probability of improvement.

Olga Laosa et al. also showed that the MIDFRAIL intervention improved frailty status and physical function in older people with T2DM at long-term follow-up (Laosa et al., 2025). Liu observed that after 12 weeks of Otago exercise intervention, the Berg Balance Scale scores in the intervention group significantly increased by 2.76 ± 0.65 from baseline. Furthermore, the intervention group showed an increase of 0.51 ± 0.63 repetitions in the 30s sit-to-stand test compared to baseline, confirming the program’s advantage in improving physical functional capacity (Yang, 2022). Alan J. Sinclair et al. note that a multicomponent intervention effectively boosts quality of life (QOL) and prevents worsening of QOL and basic activities of daily living (ADL), with high adherence required (≥93% for QOL benefits; >84.38% for Barthel Index benefits) (Sinclair et al., 2024).

The change of the SPPB score can visually demonstrate the effectiveness of the intervention measures and also predict the long-term health status. Moreover, early intervention is helpful to improve the SPPB score. Although SPPB has limitations, due to its simple operation and comprehensive assessment dimensions, it still holds an important position in quantifying the functional status of the elderly at present, providing extremely valuable reference data for clinical practice and medical research.

## 4 Exercise interventions

Exercise is the most effective intervention to slow the process of T2DM and frailty in older adults in the aging process (Millan-Domingo et al., 2024). The 2020 World Health Organization (WHO) Guidelines on Physical Activity and Sedentary Behaviour and the Physical Activity Guidelines for Americans both recommend that all older adults aged 65 and above engage in multicomponent physical activity, including balance training, aerobic exercise, and muscle-strengthening activities. When older adults are unable to meet the weekly target of 150 minutes of moderate-intensity aerobic activity due to chronic conditions, they should perform physical activity to the extent possible within their capabilities and circumstances (Bull et al., 2020; Piercy et al., 2018).

In special populations, resistance and balance training should be carried out early in acute hospitalization to prevent bed-ridden incapacitation, and low-impact exercises such as aquatic training should be chosen for patients with joint disorders. All programs need to be progressively adjusted according to individual health status such as frailty level and comorbidities to ensure safety and effectiveness.

Exercise can be categorized into six main types, resistance training, multicomponent exercise, aerobic, flexibility, balance, traditional Chinese exercises.

ME involves two or more forms of training to achieve comprehensive improvement in various aspects of the body. In the following sections we will explore the role of various types of training programs, not only as a preventative measure for frailty and T2DM, but also as an important therapeutic modality to regulate blood glucose levels and slow aging.

Helen P. Hazuda et al. found that mean HbA1c data during follow-up showed 5.93% in the intensive lifestyle intervention (ILS) group versus 6.05% in the placebo group. This result indicated that ILS was significantly better than the placebo group in terms of long-term glycemic control (Hazuda et al., 2021). Further analysis revealed that the ILS intervention may have been effective in reducing the risk of frailty by maintaining long-term glycemic stability. The data showed that the prevalence of frailty was 37% lower in the ILS group than in the placebo group, a phenomenon that showed a significant correlation with the effect of HbA1c control.

In the study by Felicia R. Simpson et al, the longitudinal mean difference between the two frailty indices (FIs) was statistically significant in the intensive lifestyle intervention (ILI) group compared with the diabetes support education (DSE) group (Simpson et al., 2020). The mean decreases in the two indices constructed on the basis of deficit accumulation were 5.8% and 5.4%, respectively, during the 8-year follow-up period. Crucially, the effectiveness of this multidomain intervention on health outcomes remained consistent regardless of participants’ baseline frailty status (Simpson et al., 2021).

In addition, the distribution of deficit status at year 8 showed that the proportion of people with deficit (FI > 0.21) in the ILI group was 39.8% and 54.5%, which was significantly lower than that in the DSE group (42.7% and 60.9%), and the between-group differences of both FIs reached the significant level (p <.001). This result suggests that multidimensional lifestyle interventions may exert a sustained inhibitory effect on the debilitating process of type 2 diabetes patients by delaying the accumulation of health deficits.

### 4.1 Resistance training

RT is one of the most important current interventions to delay frailty and T2DM. RT achieves its training effects primarily through repetitive dynamic centripetal (muscle shortening) and centrifugal (muscle lengthening) contractions in response to external loads (Smith et al., 2023). The total amount, frequency and intensity of training are three key training variables that are closely and intrinsically linked to each other, and together they determine an individual’s physiological adaptations to training and eventual exercise performance.

RT intensity as a key variable is expressed as a percentage of repetition maximum (1RM), with different intensity ranges (60%-85% 1RM) resulting in different physiological adaptations. High-intensity RT (70%-85% 1RM) has advantages over low-intensity RT in stimulating muscle protein synthesis, promoting myofiber hypertrophy, and significantly improving muscle strength.

Frequency is one of the most important factors in determining the effectiveness of training. The American College of Sports Medicine recommends 2–3 RT sessions per week. This gives the muscles sufficient time to adapt to the training load, avoids muscle damage and increased catabolism due to over-training, and maintains continuous stimulation of the muscles and metabolic system, thus ensuring the continued accumulation and enhancement of the training effect.

The total amount of training is the result of a combination of training variables. Lower training totals may not bring significant results, while too high training totals increase the risk of injury.

Increased stiffness, resulting from diminished elasticity in muscle and connective tissue, is prevalent among older adults. Therefore, resistance training programs should commence at low intensity to mitigate injury risk and progressively increase intensity over time (Tezze et al., 2023). Implement a RT program 3 times per week with 8–12 repetitions per set, starting at 20-30% of 1RM and progressing to 80% of 1RM. The program resulted in significant improvements in habitual walking speed, stair climbing ability, overall physical activity level, and increased muscle strength and explosiveness (Fragala et al., 2019).

RT has a multidimensional regulatory effect on body metabolism. This type of training can significantly promote the expression of GLUT-4 and membrane translocation, thus enhancing the efficiency of glucose uptake by myocytes; meanwhile, it can significantly enhance the sensitivity of target tissues to insulin by up-regulating the expression of insulin receptor. At the level of skeletal muscle remodeling, RT can activate protein synthesis signaling pathways such as Akt/mTOR and significantly promote myofibrillar protein synthesis. This mechanism not only reverses skeletal muscle atrophy under pathological conditions, but also increases muscle mass and strength, significantly improves somatic function indicators such as grip strength, walking speed, and comprehensive assessment parameters such as the SPPB score, and thus slows down the process of frailty. In addition, RT can reduce the level of TNF-α and other pro-inflammatory factors to alleviate inflammaging; at the same time, it can reduce the accumulation of free fatty acids, regulate lipid metabolism disorders, and form a positive cycle of metabolic improvement, thus assisting blood glucose regulation while slowing down the progress of frailty.

Lai, Xiaoxing et al. revealed that high-intensity RT induces significant muscle strength gains in frail older adults, with a robust dose-response association between training intensity and physical function improvement. Conversely, low-to-moderate-intensity RT also elicits meaningful physical function improvements while ensuring safety, tolerability, and adherence among this vulnerable population (Lai et al., 2023).

When performing RT for frail elderly patients, it is important to thoroughly assess their physical condition and tailor the training program to the individual. Since elderly frail patients often suffer from muscle strength imbalance and poor joint stability, it is important to select appropriate training equipment and devices to enhance training safety. In addition, a trainer or accompanying person should be present to supervise the whole training process and provide professional guidance and necessary assistance at any time. Compliance plays a decisive role in training effectiveness and individual health improvement. Elderly frail patients are prone to anxiety, depression and other negative emotions due to the decline of their physical functions, which may lead to the rejection of RT. Short-term and long-term goals should be set scientifically, and positive rewards should be given to enhance their sense of achievement, so as to ensure the effectiveness and sustainability of training, and ultimately realize the goal of improving the physical condition and quality of life of frail elderly patients.

### 4.2 Multicomponent exercise

The World Health Organization’s 2020 guidelines on physical activity and sedentary behavior clearly state that ME should integrate aerobic training, flexibility training, and balance training. This integration is particularly important for older adults to prevent falls and maintain functional independence.

Aerobic capacity refers to the ability of the heart and lungs to supply oxygen to the muscles, and is commonly measured as VO_2_ max. After the age of 30, this capacity gradually declines to the point where it interferes with daily activities. Endurance training can significantly increase VO_2_ peak in older adults, a key measure of frailty (Angulo et al., 2020; Villareal et al., 2004). Common forms of aerobic training include brisk walking, jogging, swimming, bicycling, and square dancing. These exercises increase lung ventilation and improve the body’s ability to utilize oxygen.

Balance training is important for preventing falls in older adults, especially those who are frail. Common forms of balance training include standing on one foot, heel-to-toe walking, standing or walking on a balance mat, and practicing Tai Chi. A study by Sherrington, Catherine et al. found that MT, which included balance training, functional exercise and RT, significantly reduced the risk of falls in older adults by 34% and reduced the proportion of people who had one or more falls by 22% (Sherrington et al., 2019).

Flexibility is defined as a joint’s range of motion (ROM), encompassing the ability to maneuver the joint through a complete ROM free of limitations and discomfort (Cai et al., 2023). Flexibility training should be carried out slowly. Long-term adherence to flexibility training, the joint mobility of the elderly will be improved, the body’s flexibility and coordination will also be enhanced.

In the multicenter clinical trial of MID-Frail, 964 frail and pre-frail elderly with T2DM were enrolled based on the Fried phenotype. The study included supervised RT, nutritional support and diabetes care. The results not only improved the patients’ somatic functioning compared to the control group, but also resulted in healthcare cost savings of €428.02 per patient per year.

ME is a comprehensive exercise strategy whose core advantage is to break through the limitations of a single training mode. In an aging society, this type of training is even more valuable. Through multifaceted neurological and muscular coordination training, it provides appropriate exercise programs for older adults with different health conditions and meets the diverse needs of the elderly population.

### 4.3 Traditional Chinese exercises

TCE include Tai Chi, Ba Duan Jin and Five Animal Play. Tai Chi emphasizes the coordination of breathing and movement. It improves body coordination and balance by increasing muscle strength, flexibility and joint mobility.

**Figure 3:**
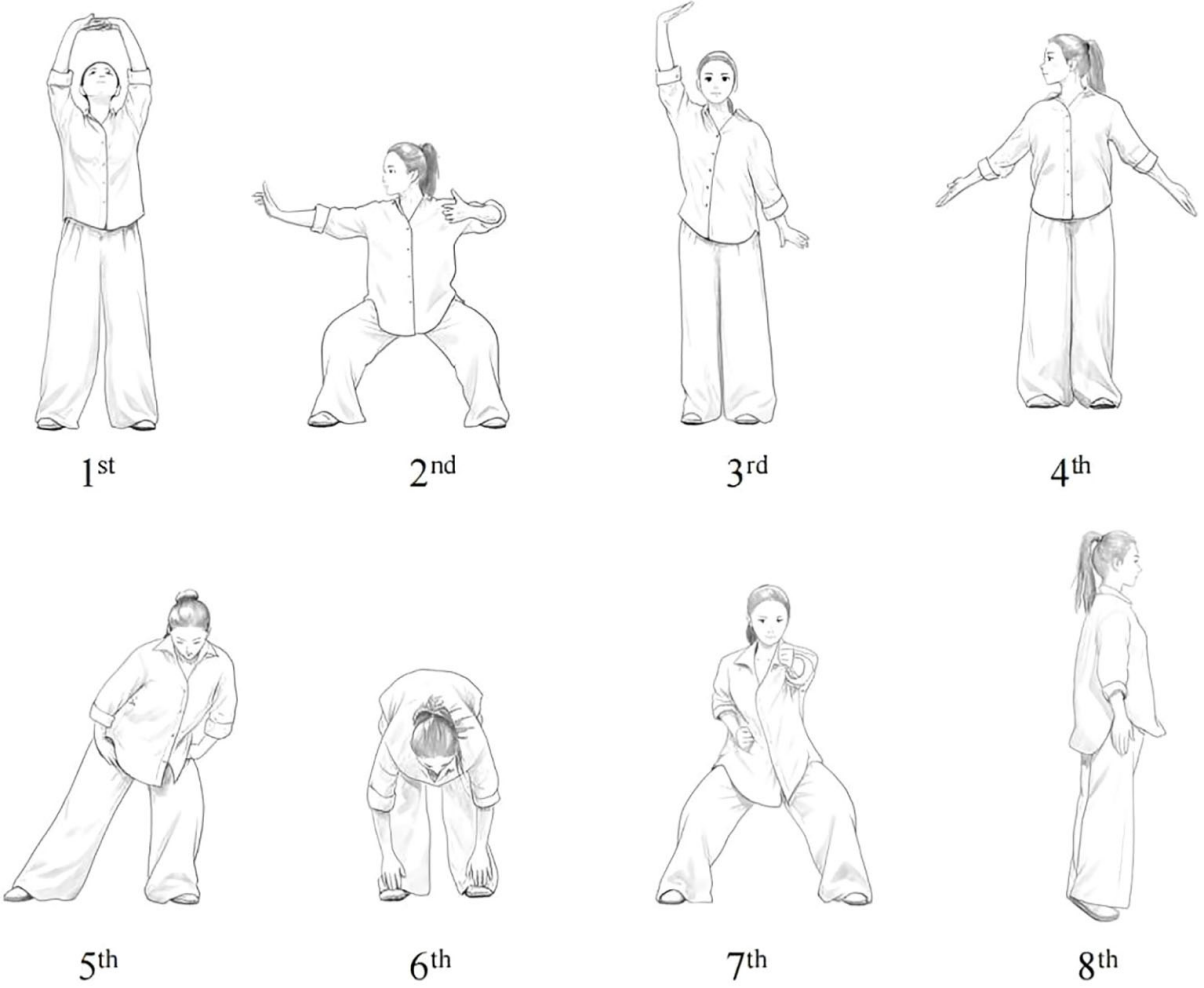
Complete movement illustration of Baduanjin.

Ba Duan Jin is simple and requires little space, making it especially suitable for the elderly and helping improve their balance to prevent falls. The Ba Duan Jin consists of eight movements, which are both relatively independent and interconnected. **Figure 3** illustrates the complete sequence of movements for the Ba Duan Jin: 1st “Raise Hands to Support the Sky” (regulate Triple Burner); 2nd “Draw the Bow Left and Right” (like shooting a vulture); 3rd “Raise One Arm to Regulate the Spleen and Stomach”; 4th “Look Backward to Relieve Fatigue and Injury”; 5th “Shake Head and Swing Hips to Clear Heart Fire”; 6th “Bend Forward to Touch Feet” (strengthen kidneys and waist); 7th “Clench Fists and Grit Teeth to Boost Strength”; and 8th “Heel Raises Behind the Back” (dispel diseases).

Five Animal Play imitates the movement characteristics and postures of five animals: the tiger, deer, bear, ape, and crane. It can enhance blood circulation and improve immunity in the elderly. When practitioners imitate these animals, it helps them relax and reduce psychological stress.

Zeng Lin et al. conducted a 12 weeks intervention study involving 70 community older adults with T2DM and frailty, who underwent structured health education sessions and regular Ba Duan Jin practice. Findings revealed significant improvements in frailty scores, glycemic control, and physical performance metrics among participants (Lin, 2024).

## 5 Discussion

This study systematically reviewed 20 RCTs to evaluate the efficacy of multicomponent exercise interventions for patients with T2DM and frailty, examining shared pathophysiological mechanisms such as chronic inflammation, insulin resistance, mitochondrial dysfunction, and oxidative stress. A multidimensional assessment was applied, encompassing frailty reversal using the FI and TFI, glycemic control measured by HbA1c, and improvement in physical function assessed with the SPPB. Based on the evidence, specific exercise prescription recommendations are provided, detailing the appropriate intensity, frequency, and duration.

Compared to previous research, this study has several advantages. This is a narrative review of exercise interventions specifically targeting individuals with comorbid T2DM and frailty. It also searched both Chinese and English databases to include more evidence, and used the rigorous Cochrane RoB 2 tool to assess study quality, making the findings more reliable. Unlike earlier studies that focused only on T2DM or frailty alone, this work examines the two conditions together. Additionally, rather than focusing on drug treatments, our study highlights the value of non-drug exercise programs. At the same time, we only included RCTs published in English and Chinese, without a systematic search of other languages. Therefore, some degree of language and publication bias may remain, and the generalizability of our findings beyond these settings should be interpreted with caution. In addition, although our inclusion criteria were set at adults aged ≥45 years, some of the included trials involved participants with a wide age range, which may have introduced age-related heterogeneity.

The value of ME intervention has been proven, but there are limitations in their practical application. In terms of long-term adherence, although exercise programs tend to motivate participants initially. As time passes, individuals have difficulty maintaining sustained participation due to accumulated exercise fatigue and declining interest. This led to a significant decrease in frequency or complete discontinuation after 12 weeks, which became a key constraint to the effectiveness of the exercise intervention. The extent to which an individual’s behavior, including taking medication as prescribed, eating a healthy diet, and making lifestyle changes, is consistent with the recommendations of healthcare professionals is defined as “adherence” (Sulwarajan et al., 2025).

Guidelines from the American College of Sports Medicine indicate that optimal programs to improve strength, cardiorespiratory fitness, agility and flexibility usually cannot be achieved through supervised outpatient sessions alone. Most people with chronic conditions or those in sports rehabilitation need a home exercise program. Technological advancements have facilitated the growth of mHealth. Smartphones are associated with apps that are widely used to track patient progress, improve access to healthcare, and manage chronic conditions (Vandelanotte et al., 2016). Studies have shown that digital interventions on smartphones are one of the effective strategies to increase motivation and long-term adherence to exercise (Svendsen et al., 2018).

The study points out that key features that enhance motivation and adherence to exercise include: the creation of rewards or incentives such as redeeming entitlements for completing goals; reminders and incentives sent via SMS, APP notifications; regular or real-time updates, feedback and progress reports; recommended workout routines, goal-setting and goal-measurement to support users’ self-recording of the exercise process, including tracking during the program and its aftermath; group classes to enhance social support, and visualization of exercise through music, animated tutorials, and gamified levels to enhance the fun of exercise (Sulwarajan et al., 2025). These strategies help users to be more proactive in staying active.

Safety considerations must be taken into account when exercising frail elderly patients. Proper warm-up, relaxation and a personalized exercise prescription are key to reducing the risk of injury. At the same time, professional guidance ensures that the exercise program is personalized to the individual’s abilities and health status. Exercise programs have been shown to have a significant effect on reducing the risk of falls in the elderly, and in particular, appropriately designed programs are effective in reducing the incidence of falls and the number of people who fall. Their effectiveness is particularly evident in programs that include balance and functional exercises or multicomponent exercise programs (Sherrington et al., 2019).

Frailty is a multifactorial syndrome, and poor nutritional status is recognized as a key factor in its pathophysiological mechanisms. Given that nutrition is an intervenable risk factor for frailty, prevention and treatment strategies should focus on implementing combined nutrition-multicomponent exercise interventions.

Sirikul, Wachiranun et al. found that ME can effectively improve physical frailty regardless of exercise duration and type. Combining ME with nutritional supplements is expected to enhance the effectiveness of reducing frailty (Sirikul et al., 2024). This synergy between exercise and nutrition can be explained, in part, by the molecular mechanisms through which nutrients influence muscle metabolism. Tezze, Caterina et al. report that nutrition, particularly proteins and amino acids, stimulates muscle protein synthesis by activating pathways such as mTOR, thereby enhancing post-exercise anabolism. Moreover, combining nutrition with exercise amplifies muscle hypertrophy, mitigates anabolic resistance in the elderly, and contributes to the preservation of muscle mass (Tezze et al., 2023).

This study holds significant clinical, public health, economic, and theoretical implications. It provides evidence-based exercise prescriptions for managing elderly patients with T2DM and frailty, offering a low-cost and scalable intervention amid global population aging. Economically, this approach demonstrates potential for substantial cost savings, as evidenced by the MID-Frail trial which reported savings of around €428 per patient annually. Theoretically, it elucidates how exercise may disrupt the T2DM-frailty cycle by modulating shared pathophysiological mechanisms. Future research should further investigate the synergistic effects of combining nutritional support with exercise interventions. Additionally, RCTs with longer follow-up periods are warranted to establish a more robust evidence base for these intervention strategies.

## 6 Conclusion

Current evidence from this narrative literature review suggests that exercise interventions, especially when integrated into multicomponent lifestyle programs, are associated with improved physical function and glycemic control in older adults with T2DM and frailty. While exercise appears to be a central driver of these benefits, the synergistic role of nutritional and educational co-interventions in many clinical trials should be acknowledged. Structured exercise programs remain a recommended strategy to potentially disrupt the T2DM-frailty vicious cycle and enhance the quality of life in this population.
