# Supplementary material for: Clinical evidence of exercise intervention in improving adults with type 2 diabetes mellitus and frailty: a narrative literature review
Source: Front Physiol. 2026 May 7;17:1791522. doi: 10.3389/fphys.2026.1791522 (PMC13189910; doi:10.3389/fphys.2026.1791522)
Supplement: Supplementary file 1 [file SupplementaryFile1.docx]

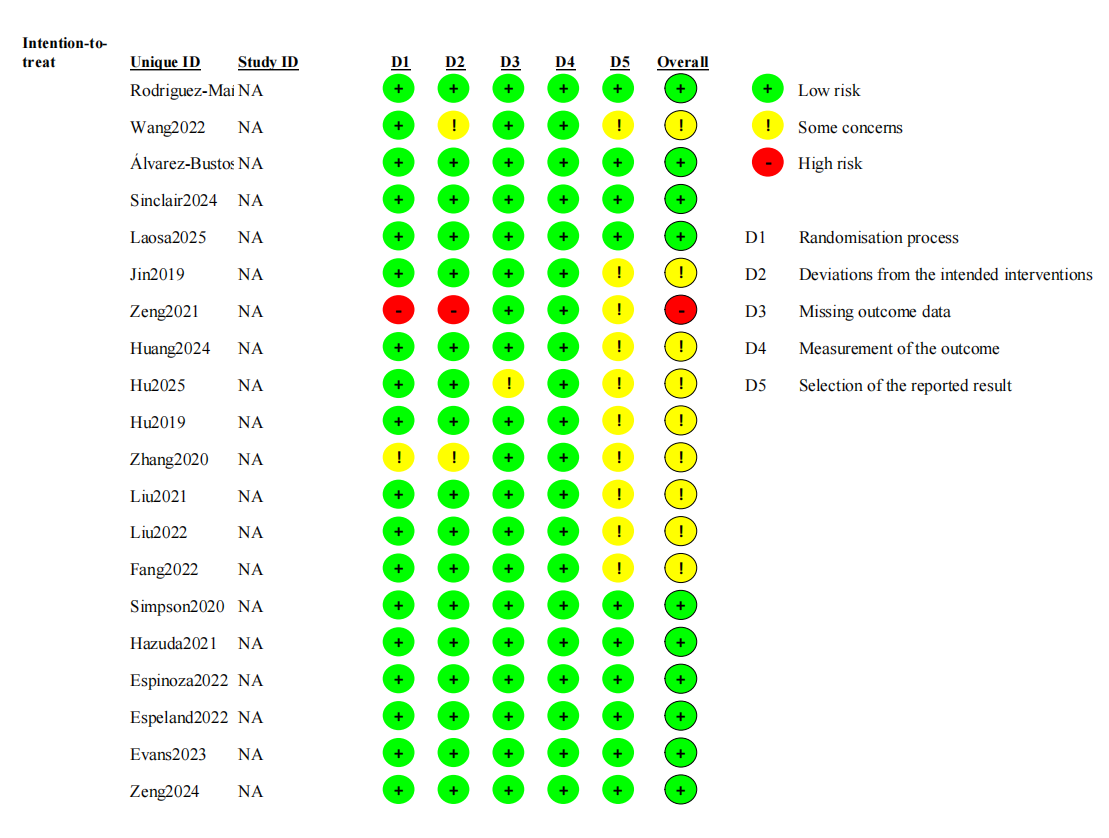


**Figure S1. Risk of bias assessment using the RoB 2 tool.**

Rodriguez-Mañas2019 and Simpson2020 were assessed as having the lowest overall risk of bias across domains. The former applied centralized cluster randomization and blinded outcome assessment, while the latter implemented advanced statistical approaches to address missing data during long-term follow-up. D1-D5 represent bias arising from the randomisation process, deviations from intended interventions, missing outcome data, measurement of the outcome, and selection of the reported result.

**Table S2 Summary of exercise intervention effects on functional and metabolic outcomes in T2DM and frailty.**

| Author and year | ① | ② | ③ | Frailty measures | Glycemic indices | Physical function |
| --- | --- | --- | --- | --- | --- | --- |
| Rodriguez-Mañas2019 |  | √ | √ |  | HbA1c attainment rate:10.8%↑ | SPPB: ↑0.85 |
| Wang2022 | √ | √ | √ | Frailty reversal rate: 85.2% | HbA1c: ↓1.26% | ESE: ↑16.82 |
| Álvarez-Bustos2024 |  |  | √ |  |  | SPPB: ↑1 |
| Sinclair2024 | √ |  | √ | Barthel worsening:↓OR=0.59 (0.37–0.93) |  | EuroQol: ↑OR=1.75 (1.20–2.54) |
| Laosa 2025 | √ |  | √ | Frailty criteria↓≥1: 51.8% |  | SPPB: ↑0.6 |
| Jin2019 | √ | √ | √ | Frailty reversal rate: 60.3% | HbA1c: ↓1.10% | BMI: ↓3.12 kg/m² |
| Zeng2021 | √ | √ | √ | Frailty reversal rate: 56% | HbA1c: ↓1.07% | BMI: ↓1.93 kg/m² |
| Huang2024 | √ | √ | √ | Physiological frailty: ↓8.55 | Fasting glucose: ↓1.61 mmol/L | QoL physical: ↓29.83 |
| Hu2025 | √ | √ | √ | Frailty score: ↓2 | Fasting glucose: ↓3.86 mmol/L | SPPB: ↑3.72 |
| Hu2019 | √ | √ | √ | Fried Frailty Phenotype symptom↓ | HbA1c: ↓1.79% | Slow gait: 90.7% → 9.3% |
| Zhang2020 | √ | √ | √ | Frailty score: ↓1 | Fasting glucose: ↓0.93 mmol/L | SPPB: ↑2 |
| Liu2021 | √ | √ | √ | Physical frailty: ↓2.49 | HbA1c: ↓2.80% | Chair stand: ↑1.52 |
| Liu2022 | √ | √ | √ | FP score: ↓1 | FBG: ↓1.21 mmol/L | BBS: ↑2.76 |
| Fang2022 | √ | √ | √ | TFI: ↓4.0 | FBG: ↓0.9mmol/L | SPPB: ↑3.1 |
| Simpson2020 | √ |  |  | FI (deficit accumulation) ↓5.8% |  |  |
| Hazuda2021 | √ | √ | √ | Frailty: ↓ OR=0.62 (0.42–0.93) | HbA1c: ↓ 0.12% | BMI: ↓ 1.0 kg/m² |
| Espinoza2022 | √ |  |  | Frailty: ↓ 0.7% (OR=0.94, p=0.60) |  |  |
| Espeland2022 | √ |  | √ | FI ↑ (greatest vs least): HR for mortality = 2.32 (1.84–2.94) |  | 400-m walk speed: ↑ 0.025 m/s |
| Evans2023 | √ |  | √ | FI-E: ↓ 0.0130 (95% CI: 0.0104-0.0156, p < 0.001) |  | Biological age: ↓ 1.67 years |
| Zeng2024 | √ | √ | √ | TFI: ↓2 | FBG: ↓1.43 mmol/L | SPPB: ↑2 |

SPPB: Short Physical Performance Battery; FI: Frailty Index; FFP: Fried Frailty Phenotype; ESE: Exercise Self-Efficacy Scale QOL: Quality of Life; HbA1c: Glycated Hemoglobin; TFI: Tilburg Frailty Indicator; FBG: Fasting Blood Glucose; BMI: Body Mass Index; BBS: **Berg Balance Scale.**

①: Frailty improvement; ②: Lower blood glucose; ③: Improved physical functions.
